# The Energy Dissipation Model of the Evolutionary Imperative

**DOI:** 10.3390/e28090948

**Published:** 2026-08-24

**Authors:** Louis N. Irwin

**Affiliations:** Department of Biological Sciences, University of Texas at El Paso, El Paso, TX 79968, USA; lirwin@utep.edu

**Keywords:** thermodynamics, complexity, geomorphology, biodiversity, forces of nature, density gradients, cosmology

## Abstract

At every level of resolution, over many orders of magnitude in time, size, and space, every aspect of the universe is constantly evolving under the pressure of the major forces of nature to resolve gradients of disparity in mass and energy. This imperative for change is channeled by two fundamental constraints: the bias of the Second Law of Thermodynamics (SLT) toward increasing entropy, and the mandate by the Principle of Least Action (PLA) that change must occur by the most direct and efficient path possible. While the SLT would seem to predict that the world would unwind rather than complicate itself, the opposite often occurs at the local level. While the evolutionary imperative drives the universe as a whole toward an ever higher level of entropy, it promotes increased local granularity and complexity to effect change in the net direction required by the SLT over the optimal path prescribed by the PLA. This provides a unifying perspective for all the complexity that astronomical and geophysical forces have created in the physical world, and that random variation and natural selection have induced in the living world―a consequence of nature’s imperative to dissipate energy as thoroughly and efficiently as possible.

## 1. Introduction

Quantum fluctuations at or immediately after the start of the explosive inflation assumed in the Standard Theory of Cosmology are thought to explain the appearance of local granularity as the cosmos unfolded. This led to dispersed pockets of concentrated matter and energy, creating density gradients subsequently resolved dynamically through the action of one or more of four fundamental forces: the strong and weak nuclear interactions, electromagnetism, or gravity [1].

These dynamic processes have been ongoing since the origin of the universe, as shifts in energy and shunts of matter regenerate new gradients of mass and energy that keep the world in a constant state of churn, resulting in the never-ending evolutionary process. The time course of these changes varies over many orders of magnitude, from the life cycle of galaxies spanning billions of years to the formation of tornadoes in a matter of minutes. Regardless of how slow or fast changes occur, evolution in the natural world never stands completely still [2]. This is what is meant by the Evolutionary Imperative.

In the physical world, long-term changes like planetary climate cycles and the formation of mountain ranges proceed from the passive operation of physical laws. In the living world, the principles of natural selection supplemented by a few other mechanisms reasonably account for the evolution of form and function. *How* evolution occurs in both the physical and living worlds is reasonably well understood.

On the other hand, *why* change does not simply lead to a steady-state equilibrium in all dynamic processes, as the Second Law of Thermodynamics (SLT) would seem to favor [3], is not obvious. In particular, the tendency for evolution to frequently lead to higher levels of organization in physical systems and greater complexity in living forms does not derive from any fundamental law of nature. I suggest here an explanation for this apparent anomaly, drawing from the insight of Arto Annila [4] and the lucid arguments of Raymond Neubauer [5] and, especially, Eric Chaisson [1,2,6,7].

Key terms in the sections to follow are used in the following way:

**Diversity** is defined as ‘the number of variable forms of a defined group, such as a particular clade of organisms.’

**Granularity** means ‘non-homogeneous.’

**Complexity** means ‘the number of parts of a system and the number of functional relationships between those parts’ [5].

**Organization** refers to ‘the arrangement of the parts of a system which can vary in degree from totally random to rigidly non-random.’ Quantitatively, it varies inversely with **entropy**.

**Energy dissipation** occurs whenever ‘the total energy of a system is reduced in the process of doing work, changing its form, being absorbed or stored, reducing entropy, or diffusing into the environment as heat or increased entropy.’

## 2. Statement of the Model

Ever since the singularity that set the cosmos in motion, the universe has unfolded in a slightly uneven manner. Matter and energy have become spontaneously more dispersed, with a consequent increase in the entropy of the universe as a whole. However, at a local level, the forces of nature have amplified granularity, resulting in the local aggregation of matter and energy, leading to enhanced concentration and complexity with a resulting decrease in entropy, contrary to the passive (spontaneous) operation of the SLT [1,8]. As long as the system is open so that energy for reducing entropy can be added, there is no violation of the Second Law. The fact that local heterogeneity persists, however, often at a greater level of complexity than its evolutionary antecedent, would appear to require the operation of another principle―one operating often in opposition to the tendency of the Second Law.

Such a principle does in fact exist. Older by a century but less well known than the SLT, it is referred to as the Principle of Least Action (PLA), or more often by mathematicians as the Principle of Stationary Action [9]. The PLA in its simplest form states that the shortest path between two points is a straight line. In dynamic terms, that means that any gradient of either mass or energy will be resolved by moving mass or transposing energy along the path of least resistance, over the shortest distance, in the least amount of time, to the fullest extent possible [10]. Energy will thus be transformed, or matter will be redistributed, in the most efficient and complete way that the system allows.

Combining the SLT and the PLA provides a comprehensive explanation for how *and* why evolution occurs over time. The SLT defines the *direction* for all dynamic events, specifying only whether the change occurs spontaneously from a state of higher to lower potential energy, or with input of energy if rising from a state of lower to higher potential energy. The PLA specifies that the *pathway* the system takes in changing from one state or place to another must proceed in the most efficient and complete way it can.

Since the middle of the previous century, beginning with Harold Blum’s seminal work, *Time’s Arrow and Evolution* [11], how the flow of energy through a system affects the organization of that system has been seen as central to the nature of change in the natural world. In particular, the inverse relationship between entropy and organization has been seen as integral to any comprehensive view of evolution. About 20 years ago, the Finnish physicist Arto Annila and his colleagues [12] began to publish a series of technical papers that merged the SLT and the PLA into the argument that change occurs through time as matter and energy resolve their concentration gradients in the direction mandated by the SLT in the most efficient and complete manner as required by the PLA. This model provides a comprehensive explanation of evolution that explains both its inevitability and its directionality. As they stated, “Hierarchically organized energy transduction … emerges naturally when it provides increased rates of energy dispersal [13].” Those concepts were argued in the form of complex mathematical equations. Charles Beck and I were inspired by this ambitious attempt to explain the salient features of evolution by writing a more accessible version of Annila’s thesis in our book, *The Evolutionary Imperative* [14], in which we reframed Annila’s argument into what we called **the energy dissipation model of the evolutionary imperative.** Our premise was that all the evolutionary changes that occur in both the abiotic and living worlds are resolutions of potential energy channeled by the PLA in the net direction required by the SLT.

This essay is offered as a succinct summary of this, with numerous examples from the physical and life sciences.

## 3. Universal Laws Driving Change

Little if anything in the universe is static for all time. On a cosmic scale, galaxies form and dissolve into one another, stars are born and die, accruing planets and moons that undergo marked changes as their climates vacillate and their liquids and atmospheres congregate, then evaporate away. Life almost certainly arises on many of those planets and moons―once underway proliferating into myriad forms in concert with vacillating climates and other environmental changes [15]. At the biological level species arise, for a brief existence in some cases but for millions of years in others, all going extinct eventually. In any single organism, metabolic activities enable growth, differentiation, motion of materials into and out of cells, and movement of the body, in the case of animals. Hearts force fluid through vessels, antibodies engulf pathogens, and organs autonomously carry out specialized functions. In short, the whole world changes over time, rapidly in some cases, much slower in others, but everything in it *evolves*.

The rearrangement of matter and redistribution of energy that constitute the ongoing dynamic changes throughout the universe can be traced to the four fundamental forces mentioned previously [16]. The **strong** force binds subatomic particles together in the nucleus of atoms. The **weak** interaction accounts for radioactive decay, emitting subatomic particles like electrons, positrons, and neutrinos. **Electromagnetism** derives from the behavior of charged particles, manifested as the force of magnetic attraction or repulsion, electricity, light, and radiant heat. **Gravity** bends space–time in the presence of mass, inversely to the square of the distance over which it acts. The strong and weak forces are very strong but act over atomic dimensions, while electromagnetism and gravity are much weaker but act over cosmic distances. Collectively, these forces account for every change that takes place in the universe. Thus, they provide the fundamental force for evolutionary change in both the abiotic and biotic worlds.

All the dynamic changes that keep the universe in action involve the conversion of energy from one form to another, or the redistribution of matter through the expenditure of energy. The local granularity of matter and energy generates the gradients on which the forces of nature operate to bring about those dynamic changes. But the forces of nature are not unconstrained. One law and one principle are particularly important in specifying how the fundamental forces of nature operate. One, the **SLT**, specifies whether energy is consumed or emitted, depending on the direction that change takes. The other, the **PLA**, determines the path that change will take in resolving density gradients of mass or energy.

### 3.1. Second Law of Thermodynamics

Thermodynamics matured as a science during the industrial revolution principally among scientists, engineers, and industrialists concerned with measuring the maximal work extractable from steam engines and the efficiency of machinery [17]. The first and second laws of thermodynamics were codified in the mid-19th century. The First Law states the widely acknowledged supposition that the total amount of energy in the universe is constant, and that matter and energy, while interconvertible, can thus neither be created nor destroyed in their totality [18].

For the purposes of this article, the Second Law is more directly applicable. William Thompson (Lord Kelvin), who is widely credited with formulation of the SLT [19] expressed it thusly: “I believe the tendency in the material world is for motion to become diffused … that no physical action can ever restore the heat emitted from the sun …”. Another useful way of stating the SLT is the following:

Whenever energy is transformed, not all of it can be used for useful work, because some of it is dissipated as heat and the process of increasing the random arrangement of matter.

A simple and straightforward mathematical expression for the SLT is given by the free energy equation,

Δ*F* = Δ*U* − *T*Δ*S,* (1)

where F is the free energy available for doing work at a constant temperature T, U is the total internal energy of the system, and S is entropy, or the degree of disorder in the system [3].

Work is the use of energy to effect material change. When a system moves from a state of higher to lower potential, the internal energy of the system (Δ*U*) goes down. “Potential” can be thought of as concentration of either matter or energy. The difference between a higher and a lower potential defines a gradient, so the SLT says that matter diffuses and energy flows always from regions of higher to lower concentration, when they do so spontaneously. This is true even if the internal energy remains constant, with the dispersal of heat or increased disorder (entropy) within the system (Δ*S* is positive) being the only result. When work is used to raise the potential energy of the system, or decrease the random arrangement of its components (Δ*S* is negative), energy must be added to the system. To the extent that the added energy is used to reduce the randomness of the system’s components, that energy does not add to the potential energy of the system. In either case, the net effect is that energy usable for work is dissipated.

By way of illustration, consider the following examples:

1. A boulder rolls downhill of its own accord once restraint to its motion is removed, but never rises uphill spontaneously. Higher potential energy due to elevation is converted to kinetic energy as objects fall down the gradient of gravitational attraction.
2. A sugar cube dropped into a cup of coffee dissolves and disperses throughout the volume of the cup. This occurs spontaneously as the molecules of sugar move from a compact, highly ordered (low entropy) aggregate to more randomly dispersed molecules with higher entropy. Little energy is released in this case; most of the change goes into increasing the entropy of the sugar molecules.
3. A hot bowl of soup will cool to room temperature spontaneously. A cold bowl of soup, however, will not rise above the temperature of its surroundings of its own accord, even though there is plenty of heat energy in the combined air molecules surrounding the soup to heat it if somehow that heat could be gathered in from its dispersed location and concentrated into the soup bowl.
4. Construction of a building requires a great input of energy, because so much material has to be arranged from highly dispersed origins into a compact and geometrically rigid structure. If initiated by a few well-placed charges of dynamite, the building will collapse spontaneously; however, the individual bricks and girders will never reassemble themselves into an intact building without an input of labor.

### 3.2. Principle of Least Action

If a ball is dropped from the balcony of an upper story in an apartment building, it will fall to the ground in a straight line. If dropped inside the stairwell from an upper floor, it will bounce down the stairs along the steepest trajectory possible within the confining walls of the stairwell. If the ball is dropped at the top of a long hill, it will roll to the bottom along the steepest path it can find around the boulders, bumps, and trees along the way. Yet no fundamental law of nature requires the ball to fall in a straight line from the balcony or take the most direct path possible down the stairs or the hill. Intuitively we know that the ball will take the shortest unobstructed path it can in response to the force of gravity, but intuition was not a satisfactory explanation for the scientists of the 18th and 19th centuries who gradually unveiled a more rigorous understanding of why the displacement of matter or the flow of energy takes place over either the shortest distance or the least time it can, depending on whether the pathway for those changes is measured in terms of distance or time. The formal doctrine has come to be known as the **Principle of Least Action (PLA)**. Contemporary mathematicians prefer to call it the Principle of Stationary Action, to convey the concept that the actual path that nature takes in moving matter or transforming energy coincides with the mathematically optimal path, hence the actual trajectory is “stationary” relative to the optimal path [10].

The earliest formal description of the PLA is generally attributed to Pierre-Louis Moreau de Maupertuis (1698–1759), a French mathematician and biologist [20]. The renowned Swiss mathematician, Leonhard Euler (1717–1783) focused on applying the PLA to the displacement of mass, stating that a given amount of energy will be expended over the shortest distance possible to get an object from one point to another. Assuming that the amount of energy for moving a mass over a given distance (work) is constant, this amounts to saying that a given amount of work will be performed in a way that consumes the least amount of energy necessary. Stated another way, it says that the amount of work performed will be maximized per unit of energy consumed. In this formulation, energy is transformed in a maximally efficient way.

The French–Italian mathematician Joseph-Louis Lagrange (1736–1813) worked with Euler to produce a version of the principle in which the “action (A)” in simple cases is the change from potential to kinetic energy integrated over time [10].

(2) A=∫(K−P)dt

where K minus P is the difference between kinetic and potential energy. This means that energy is transformed to the maximum extent possible per unit of time for a given change in the difference between kinetic and potential energy, i.e., until energy conversion is maximally complete. From this perspective, the release of potential energy per unit of time is maximized to the extent allowed by the system. Stated another way, a system will transform the most energy it can within the time available. The stone will roll as far down the hill as it can, transforming the potential energy of height into the kinetic energy of motion, as fast as it can within the limits allowed by the force of gravity and the resistance it encounters along the way. This can be described as energy transformation that is maximally complete.

Some systems use energy more efficiently, while others consume or conserve it more completely; and the two are not always mutually compatible. Tradeoffs are sometimes necessary. The PLA favors both, with emphasis on whichever facet of energy usage is required by the system in question. If travel occurs with the same velocity regardless of the path taken, the path between two points that is the shortest in distance also takes the least amount of time to cover. Thus, the PLA requires the displacement of matter or the flow of energy to occur over either the shortest path available or the shortest time possible. The term “effective” can be used when referring to energy transformations that are either maximally efficient or maximally complete, or both.

It should be noted that this is a simplified interpretation of “least” or “stationary” action. A full and rigorous presentation of the PLA is provided in the publications of Annila [13,21,22]. An analogous concept that started gaining adherents in the latter part of the previous century is the Maximum Entropy Production Principle (MEPP) [23]. Stated simply, the MEPP says that in local systems far from thermodynamic equilibrium, stable dissipative structures will arise that maximize an increase in entropy while keeping the entropy of the local structure low by discharging increased entropy into a surrounding reservoir [24]. A dissipative system or structure is a thermodynamically open system that operates far from thermodynamic equilibrium and exchanges energy, matter, and information with the external environment [25,26]. The MEPP provides similar explanatory power to that of the PLA, and is particularly effective in dealing with biological phenomena which are self-organizing, dissipative structures that are locally stable thermodynamically but are far from thermodynamic equilibrium with the larger environment [27]. Since energy dissipation rather than entropy maximization is the emphasis in this essay, in most cases reference will focus on the PLA, but in many cases the MEPP would also provide a useful framework.

### 3.3. Contrasting Nature of SLT and PLA

The SLT provides the impetus for change, by favoring the tendency for density gradients to resolve spontaneously with an increase in entropy and decrease in total internal energy that is irreversible. A change that increases the magnitude of the gradient can occur only if new energy is consumed. By contrast, the PLA specifies the path but not the direction of change in magnitude that gradients bring about in the process of creating change. The PLA is indifferent to whether entropy is increased or decreased by the change.

All the changes that occur in the natural world are channeled by these two overarching principles―the SLT mandating the uptake or release of energy, depending on the thermodynamic direction of change, and the PLA defining the optimal path irrespective of its direction. The two principles are neither complimentary nor parallel. Organisms grow in size and complexity by consuming energy from the environment, as mandated by the SLT. In doing so they use metabolic pathways that have been shaped for efficiency by natural selection in accordance with the PLA to degrade the energy from sunlight in the process of conserving potential chemical energy within the organism. As long as it lives, the organism resists the SLT mandate to spontaneously expire by a continual dissipation of energy, but when it dies its entropy increases to its ultimate point of dissolution, again along a path determined by the PLA.

The operation of these two profound principles of physical science provides a unifying framework for all the organization that we see in the universe, and most significantly all of the evolutionary changes that have been brought about. Together they constitute what here is referred to as **the energy dissipation model of the evolutionary imperative**.

## 4. Change in the Physical World

Most cosmologists hold the view that the four principal forces of nature separated from one another within the first microsecond of the explosive expansion of space-time that marked the origin of our current universe around 13.7 billion years ago [28]. It took a few hundred thousand years, though, for the universe to expand and cool in accordance with the SLT sufficiently for protons and neutrons to capture electrons and form the first atoms. While most of the first atoms were the simplest possible―hydrogen, with one proton and one electron―the strong nuclear force enabled two protons and two neutrons to bind together and capture two electrons to form helium in a ratio to hydrogen of about 1:10. At a much lower frequency, three protons and four neutrons came together, attracting three electrons to form lithium. Hydrogen, helium, and lithium were the first atoms in the beginning of our universe.

### 4.1. Evolution of Stars and Planetary Systems

Due to inhomogeneities in the young cosmos, presumably deriving from quantum fluctuations during its beginning, the density of those earliest atoms was slightly greater at some points than others [16]. Under the influence of gravity, atoms began to congregate, with greater concentrations forming where the density of atoms was greater. These local disparities in the accumulation of mass set up positive feedback loops in which local regions with greater mass became progressively more massive, drawing in less densely packed atoms. In time, the accumulation of hydrogen became so massive that gravity was strong enough to force those atoms to start fusing into helium. When the protons from four hydrogen nuclei (^1^H) fused into a single helium nucleus of two protons (^2^He) and two neutrons, the total mass of the helium nucleus was slightly less than the mass of the four hydrogen protons. The difference was discharged in the form of radiation (neutrinos, positrons, and photons). Thus, the emission of light and other forms of solar radiation began, and a star was born.

In stars massive enough, the force of gravity pushing the three original atoms together generated heavier elements [29], like carbon (3 × ^2^He → ^6^C), nitrogen (^3^Li + ^3^Li + ^1^H → ^7^N), oxygen (4 × ^2^He → ^8^O), silicon (2 × ^7^N → ^14^Si), phosphorus (^7^N + ^8^O → ^15^P) and sulfur (2 × ^8^O → ^16^S). These newer elements formed concentric rings of increasingly massive elements outward from the star’s center of gravity, reflecting increased local complexity driven by the mandate of the PLA for smaller atomic nuclei to descend down their steepest density gradient toward capture by the strong nuclear force into larger, more complex atomic nuclei. In stars much larger than our sun, which were more plentiful in the early phase of the universe, much heavier elements were formed up to iron (^26^Fe), the heaviest element that could be created by nuclear fusion. These larger stars would grow so massive that gravity crushed all the atoms together to the point that repulsive forces overcame the force of gravity and the star exploded in a supernova that spewed its contents out into space. The force of this explosion was sufficient to generate some elements even heavier than iron, and all the residue of the supernova provided dust that would congeal into a new round of star formation [30].

While most of the atoms in space were brought together by gravitational condensation into stars, at the outer reaches of each star’s gravity well, local concentrations of matter clumped together to form planets that started circling around the stars in orbits ranging from near circular to very eccentric. The positions and kinetic energy of the planets in their early stages of formation assumed the optimum orbit allowed by the conditions of their formation in accordance with the PLA. In a similar manner, smaller lumps of matter formed moons around the planets, or in some cases passing protomoons were captured in orbit around the planets, all circling around their central planet, just as the planets orbited their central stars [29]. All the planets and moons of sufficient mass assumed globular shapes, like the stars that held them, minimizing the distance from every point on their surface from the center of gravity. Thus, recurrent cycles of supernovae produced the chaotic dispersal of matter that increased entropy due to the SLT when the stars exploded spontaneously. Then, the force of gravity emplaced the motion of stars, orbited by planets encircled by moons in organized patterns at lower levels of entropy than the dispersed atoms and molecules from which they were constituted, as required by the PLA.

### 4.2. Evolution of Planetary Topography

By the time a planet had accreted close to its ultimate mass, the kinetic energy of its bombardment by meteorites and smaller planetoids had delivered a great deal of kinetic energy that was stored in the newly formed planet’s interior. This thermal reservoir, along with the energy released from the weak nuclear forces by radioactive decay of heavier elements near the planet’s core, resulted in a strong gradient of differential heat between the core of the planet and its surface. This in turn provided the geothermal forces that led more solid continental plates which floated at the surface on the molten interiors to collide or scrape across or beneath one another, causing earthquakes, volcanic eruptions and geysers in the short term and the creation of mountain ranges over longer periods of time.

The eruption of a volcano or geyser emits a spontaneous release of energy resolving the gradient between the hot, high-pressure interior and the cold, low-pressure surface, dispersing soil, magma, and water, leading to an increase in entropy as required by the SLT. The formation of a new mountain range from colliding plates shows how a portion of the spontaneous energy released in resolving one gradient can create a new one. Orogeny (mountain building) establishes a new potential energy gradient between the increased elevation of the mountain and the lower surrounding countryside. In time, the dynamic action of wind and rain will dislodge rocks and soil from the higher elevations, dispersing material under gravitational force down the side of the mountain along the path of least resistance as required by the PLA. Boulders break into rocks, and rocks become sand as they wind their way to the sea.

**Figure 1:**
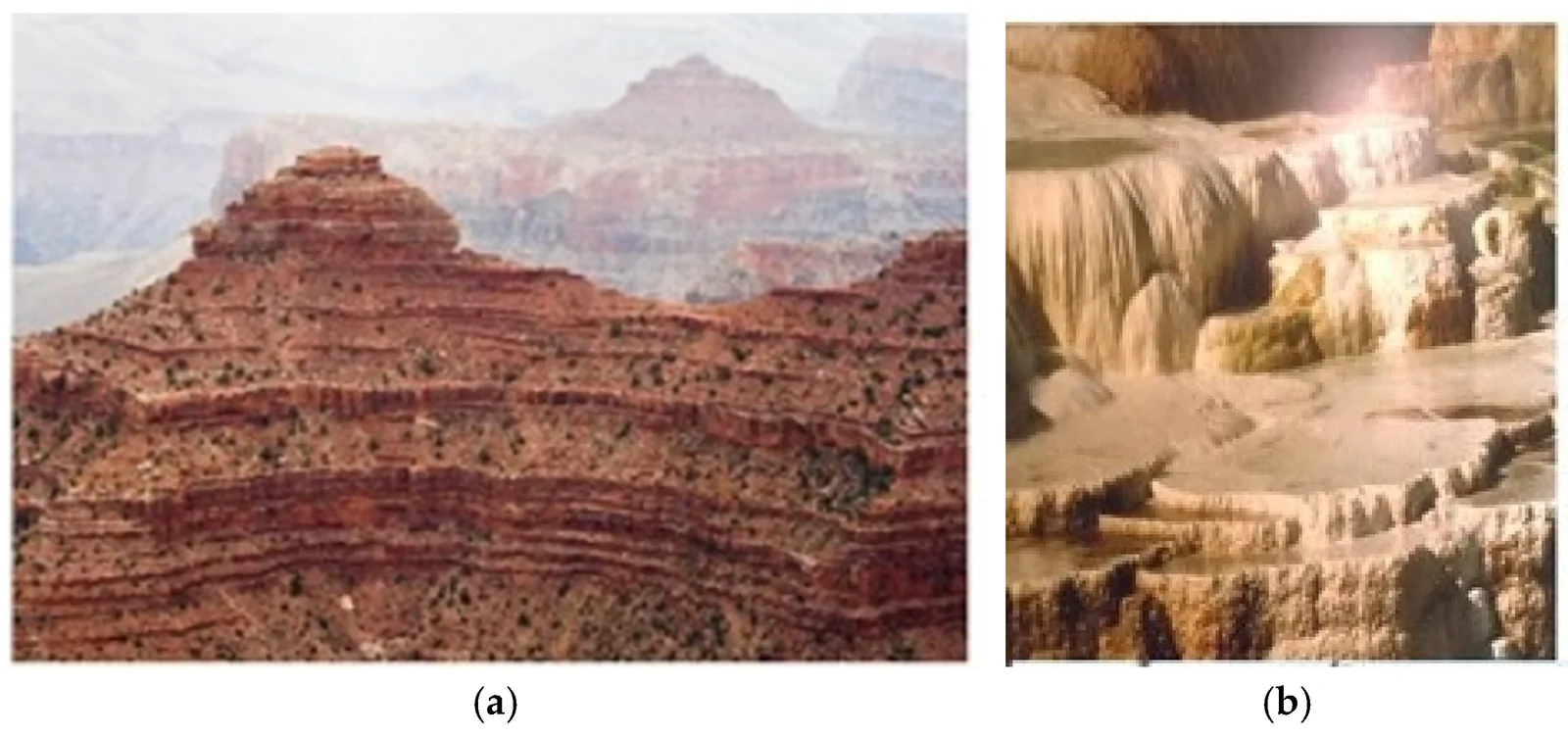
Order in nature. (**a**) Erosion reveals the sedimentary formations laid down by successive waves of infill into the great inland sea of the Mesozoic in what is now North America. *Photography in Theodore Roosevelt National Park by Laura Thomas, National Park Service*. (**b**) Gravity and the PLA combine to create complexly arrayed and ordered pools of water, as walls formed from calcium carbonate precipitating out of hot springs form a series of containers for water that spreads to its lowest possible level of potential energy within each container. *Photography of Yellowstone National Park, Wyoming, by Louis Irwin*.

The resolution of all these gradients over time results in a net loss of energy capable of doing work, since a portion of the energy turns into heat and disperses matter, leading to a net increase in entropy. Energy is thus dissipated in the process, as completely as possible according to the PLA. Yet at the local level, the consequences of these dynamic actions are not at all random, leading in some cases to highly organized structures. This is seen in the sedimentary layers of soil found on every continent where natural erosion or human road cuts have revealed a pattern of sequential deposits characterized by the environmental conditions at the time each layer was deposited (Figure 1a). Volcanic ash colored some layers gray, while high levels of oxidation at a different age turned minerals red. Dense vegetation from millions of years ago are turned into seams of coal or liquified hydrocarbons, depending on depth, pressure, and the nature of what had been living organisms. The tendency of water to seek the lowest level possible within a given container leads to an ordered cascade of pools in some cases (Figure 1b), or streams that turn to rivers that gouge out canyons in others.

**Figure 2:**
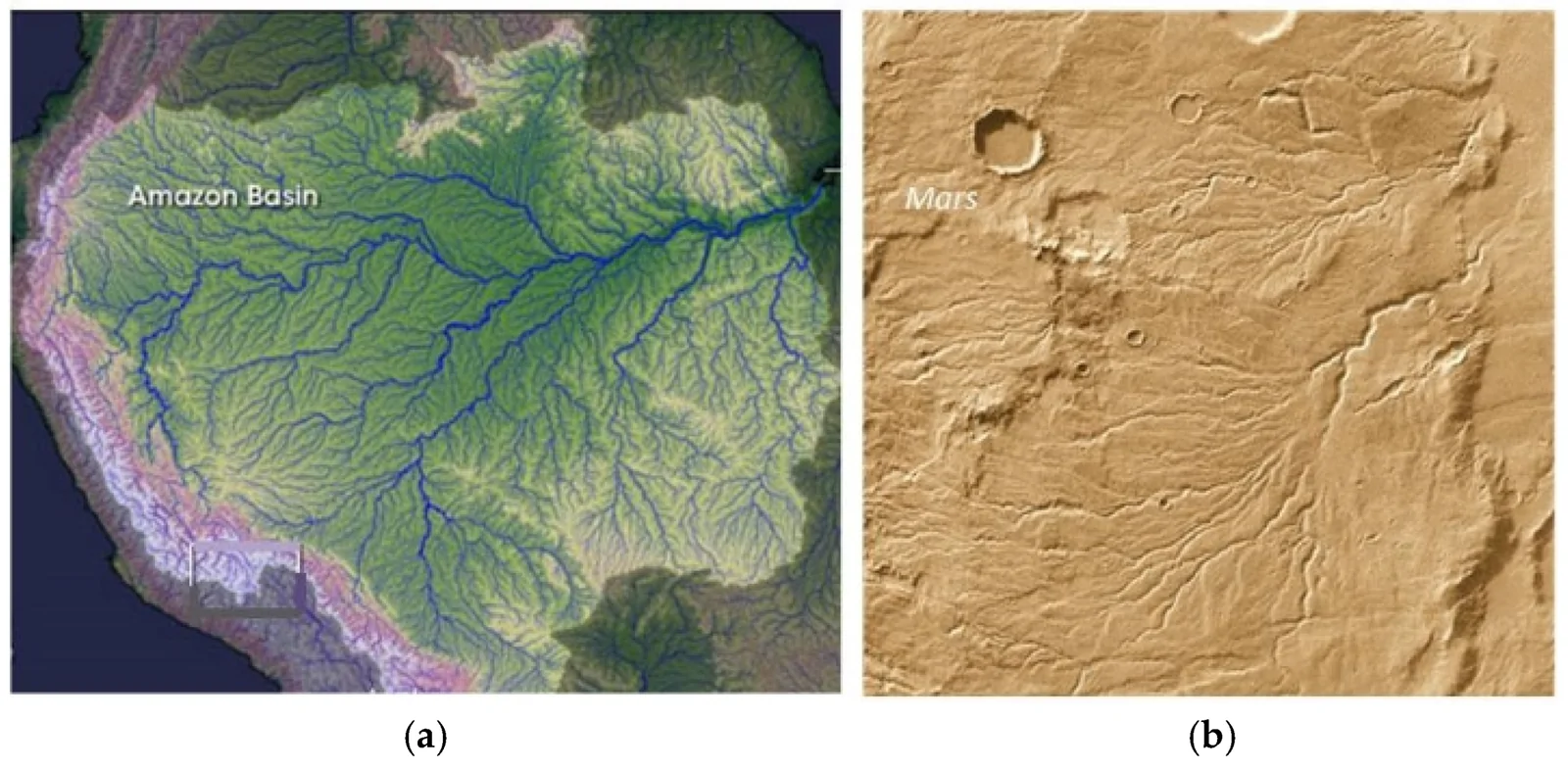
Drainage basins. Water flows downhill, along the optimal paths available to it under local circumstances, whether in the Amazon basin (**a**) or on Mars (**b**). *Credit*: (**a**) *NASA Earth Observatory*; (**b**) *NASA/JPL/Arizona State University*.

Water that falls in small volumes as raindrops over large areas generates a highly ordered dendritic pattern of branches that converge into growing volumes in channels that follow the least obstructive path to the lowest elevation they can reach at sea level―on Mars as on Earth (Figure 2), and wherever else the shape of the land defines the path of flow.

### 4.3. Weather and Climate

Weather refers to the dynamic activity of the troposphere (the lowest level of the atmosphere) in concert with the water cycle in a local area. This includes the wind, rain, snow, ice, hail, storms, drought, and daily cycles of heating and cooling. Like all the other changes in the physical world, they trace to the same fundamental forces of nature, framed as always by the unifying perspective of the SLT and PLA.

Weather dynamics are driven by solar radiation (produced by the weak interaction in the nuclear fusion process), but are modulated by conditions on the planetary surface. When air over the surface is warmed by solar insolation, it expands, reducing its density and exerting less pressure at the surface. When air cools, its density goes up, and pressure increases. The difference in atmospheric pressure between adjacent highs and lows constitutes the gradient that provides the motive force for movement of air, or wind. Morning and evening breezes reflect the gradients caused by warming air as the sun rises, and cooling air as the sun sets.

Stronger winds are generated by stronger gradients. Over the continental land masses, far from the thermal buffering of the ocean, temperatures rise much higher in the summer and drop much lower in the winter. In the mid latitudes of both hemispheres where seasonal variations in temperature are significant, the transition from winter to summer is occasioned by the most extreme clashes between cold and warmer masses of air, too often resulting in tornadoes. In the tropics, prolonged periods of warmth cause extreme low-pressure cells to form over the ocean near the end of summer. As the warm air rises, it starts to rotate due to the Earth’s rotation plus the same conservation of angular momentum that occurs whenever matter is moved due to any disparity in the density of mass or energy. Except this rotation is more local and more rapid by orders of magnitude, resulting in a hurricane.

Precipitation occurs because the ability of air to hold water vapor depends on its temperature. Warm air holds more water, while cold air causes it to condense and fall from the sky as rain, hail, or snow. As water flows from wherever it falls or melts to the lowest elevation it can reach, it carries with it eroded soil from the land through which it passes. The energy from the sun is thus dissipated through the evaporation of water, the force of wind set in motion by differential gradients of atmospheric pressure, and the inevitable tendency of matter to scatter in an increasingly random manner when spontaneously dispersed by the forces of nature.

Climate is the summation of atmospheric activity and related environmental conditions, including solar radiation, temperature, humidity, precipitation, and wind averaged over a longer period of time. Tropical climates are concentrated nearer the equator, where sunlight strikes the Earth at a more consistent angle closer to the perpendicular. Temperature oscillations with the seasons are greater at mid-latitudes where the intensity of sunlight varies with the seasons over a wider range, due to tilt in the Earth’s axis, which results in stronger, more direct sunlight when a hemisphere is tilted toward the sun in summer, and weaker, less direct sunlight when the tilt is away from the sun in winter. Because the Earth wobbles on its axis over long-term cycles―every 40,000 years, the tilt reaches 24.5 degrees―the average temperature of the planet drifts up and down over these longer time intervals. Ultimately, weather and climate are both functions of variable degrees of solar insolation, which is a function of orbital mechanics determined by gravitational interactions between the Earth and the sun. The weather and climate on other worlds, where the orbital mechanics are very different, can be expected to differ greatly from that of Earth.

### 4.4. Nature of Change in Physical World

Regardless of the timescale or magnitude of changes in the physical world, all of them can be traced to the fundamental forces of nature, which create gradients of mass and potential energy, then resolve those gradients in accordance with the SLT, along the specific pathways that the PLA demands. In requiring that matter move and energy flow along the steepest, straightest paths possible, however, the PLA imposes a sense of order that frequently manifests itself as growing complexity. Typical examples are the symmetrical beauty of a shield volcano, the ordered layering of sedimentary rock, the stepwise descent of static pools, and the meandering but maximally efficient path that water takes in flowing through dendritic patterns of channels that converge increasingly to the discharge of a maximum volume of water at the lowest elevation available.

Every evolutionary event in the physical world, however, is independent of preceding events, save for the contingency of local and pre-existing conditions. This is a critical point that deserves emphasis. Each occurrence of a physical change is essentially an independent event, bearing no necessary relationship to similar events that have preceded it. Mountains come and go, but no two mountains are the same. While tornados and hurricanes are highly organized, the microstructure of each one is totally unique―no pre-existing storm dictates the detailed structure of any that follows. Information from one event is not carried over to the next. The same cannot be said for the evolution of life, with dramatic consequences, as the next section reveals.

## 5. Change in the Living World

There is virtually universal agreement on three characteristics of life that are relevant to the hypothesis argued here. They are that living organisms (1) encompass an internal environment at a lower state of entropy in thermodynamic disequilibrium with the external environment, which (2) consume energy from the exterior to maintain their highly ordered interior and carry out intrinsic activity, and (3) are the product of near-exact replication of a predecessor by using genetic information through a process that is totally autonomous [31].

This view of life shifts the basis of change in the natural world from physical forces to chemical interactions, and alters the focus from very large units (e.g., planets, mountains, hurricanes) to a much smaller scale (e.g., organisms, cells, molecules), with radical changes in time course (from billions of years for planets, millions for mountain building, and months for seasons, to years for organisms, weeks for individual cells, and microseconds for metabolic reactions). And unlike the physical world, the living world relies on the continuity of pre-existing form and function through replication.

Life is sustained through dynamic processes at the molecular level for which the specificity of interactions is critical. This depends on macromolecular building blocks and enzymes that interact with small metabolites and each other. Macromolecules are built up by constructing chemical variations along a backbone chain of mostly carbon atoms with intermittent bridges of nitrogen and oxygen [32]. Construction of such molecules requires an input of energy, since their greater complexity is lower in entropy than the collection of smaller constituents of which they are built.

Energy dissipation was both the cause and effect of increasing chemical complexity at the origin of life. The construction of larger, more complex molecules from smaller, simpler ones was inherently an energy-consuming process. In addition, the synthesis of larger, more complex biomolecules serving as enzymatic catalysts, transporters, and other forms of dynamic metabolites promoted more energy-consuming metabolic activities such as energy mobilization and storage, ionic and molecular transport, cellular and subcellular construction, and intracellular signaling. Once carboxylic acids or other hydrophobic molecules became abundant enough, self-forming vesicles enclosing protometabolic activity would have become possible [27]. Eventual enclosure of these various processes and capabilities into protocells provided a distinct separation between the contents of the highly organized, low entropy interior and the higher entropy of the external environment, setting up the thermodynamic disequilibrium characteristic of all living systems.

Reactions inside these protocells would have proceeded at a much higher rate than in the unsequestered surrounding environment, partly because more frequent reactions were facilitated by the greater concentration of reactants inside the vesicles, and by the growing repertoire of biomolecules with catalytic activity. Protocells thus would have provided a much higher rate and more efficient utilization of energy than the surrounding environment, increasing the net entropy of the protocells and their environment faster and to a greater extent as mandated by the PLA, with the direction of energy flow determined by the SLT.

The emergence of protometabolism is a watershed event that presumably continues to occur on at least some planetary bodies wherever suitable conditions for it appear [15,33,34]. The energy transformations miniaturized within the incipient cells brings the preponderance of energy flow down to the molecular level. At that point, more energy per unit mass is being degraded by the interaction of molecules than the erosion of mountains. Physics gives way to chemistry as the prevalent venue for energy dissipation in living systems, bringing miniaturization and precision of information to the forefront in the evolutionary process.

### 5.1. Origin of Life

Most hypotheses about the origin of life assume that it emerged from its non-living surroundings by an extremely slow, erratic, and imprecise process that was widespread (possibly global), redundant, and drawn out over millions of years [35]. There is logically no reason why life could not have originated exclusively at a single place and time. However, the complexity of form and function required at all levels of living systems makes the gradual refinement of erratic processes in recurrent forward and reverse steps, integrated over a long period of time and space, seem more plausible.

Organic building blocks for the biomolecules of living cells—especially numerous amino acids and fatty acids—are delivered regularly from comets and stardust to planetary surfaces by meteorites [36,37,38]. In addition, it is widely assumed that the monomers (amino acids, carboxylic acids, nucleosides, and sugars) that are polymerized into peptides and proteins, fatty acids, nucleic acids, and polysaccharides that constitute the structures and active metabolites of living cells were assembled from simpler compounds like ammonia (NH_3_), carbon dioxide (CO_2_), hydrogen cyanide (HCN), nitrous oxide (NO_2_), and sulfur dioxide (SO_2_) during early stages of planetary history, with an input of naturally available energy such as lightning [39,40,41,42].

For life to sustain itself, a mechanism for replication is essential. Life on Earth today encodes the information necessary for all living processes, including the ability to self-replicate, in long double-stranded molecules of deoxyribonucleic acid (DNA). But almost no biologist believes that DNA was the first informational molecule with genetic capabilities. Ribonucleic acid (RNA) operates through shorter, single-stranded segments, has all the informational capacity that DNA has, and furthermore has catalytic properties. A widely held view, therefore, is that an “RNA World” must have preceded the age of DNA-based cellular control [43,44]. However, RNA itself is a complicated molecule and seems unlikely to have emerged in its current form from the primitive metabolic environment that must have characterized the earliest, incipient forms of replication. A more primitive mode of replication is almost sure to have preceded the process that took hold in the RNA World [45,46]. Persuasive arguments have been made that RNA is the evolutionary remnant of an earlier inorganic template, serving a cruder alignment and catalytic function found in the pre-life era. Minerals composed of reproducible crystalline architecture could have served such a non-living template function [47], and the metallic atoms that are co-factors in enzymes today may be descendant reflections of catalysis performed by the naked atoms before the dawn of life. Graham [48] provided the most complete scenario for evolution of replication and catalysis originating from crystalline minerals.

A reasonable question is whether any replicating informational molecule could have arisen before a fairly complex repertoire of metabolic reactions for synthesizing it had evolved. This question, in oversimplified form, is as follows: What came first―replication or metabolism? The trivial answer is that any replication requires a metabolic process of some sort, but the persistence of any particular metabolic pathway―and reliable replication depends on the persistence of particular metabolic pathways―requires some means of replication. It is difficult to see how one could emerge without the other. Therefore, while arguments for replication first [49,50], on one hand, and metabolism first [51], on the other, have been made, those models that envision coevolution of metabolism and replication seem more likely to represent the actual history of the origin of life [14,33,52]. The centrality of the evolution of metabolism to the appearance of life gives credence to the thesis that energy mobilization under the SLT and PLA was the guiding and driving force behind the origin of life. This assumption also fits well within the framework of the MEPP [24].

### 5.2. Evolution of Life

Erwin Schroedinger [53] is generally credited with initiating the modern trend toward viewing life through a thermodynamic lens by noting that organisms maintain the living state by drawing negative entropy (negentropy) from their environments. That trend can be traced earlier to Lawrence Henderson [54] who wrote that “… peculiar and unsuspected relationships exist between the properties of matter and the phenomena of life.” Since energy is obviously integral to living processes, special attention has focused on the flow of energy and its utilization in modern biology. The importance of energy processing was suggested early in the 20th century by Alfred Lotka [55] who wrote that “natural selection will so operate as to increase the total mass of the organic system, to increase the rate of circulation of matter through the system, and *to increase the total energy flux through the system* (italics added)…”. For decades, Harold Morowitz [35,56] provided compelling arguments that biological organization is driven by the flow of energy that keeps living systems at a low level of entropy. Eric Chaisson [1,2], like many others, has linked the SLT explicitly to organic evolution, arguing that the ongoing expansion of the universe in accordance with the SLT is the ultimate engine for the evolution of life. He has advanced the concept of “energy rate density”―the amount of energy transformed per unit of time and mass―as a comparative metric for the degree to which energy is degraded by different systems [6]. Arto Annila and his colleagues [21,22] have introduced the PLA as a critical component of their formal and sophisticated treatment of evolution in thermodynamic terms. They have modeled evolution as proceeding down pathways with the steepest descent in energy. This frequently entails a maximum increase in entropy, as modeled within the MEPP framework [24] as well.

#### 5.2.1. Emergence of Self-Perpetuation and Natural Selection

Assuming that replication was guided initially by an external template of some sort, this process cannot be said to be autonomous until a mechanism emerged that could operate independent of the external environment. Therefore, the most plausible models for the origin of autonomous replication envision that metabolism, replication, differentiation and division coevolved in a way that the protocells could divide into “daughter” descendants capable of carrying out all the metabolic activities of the parental cell. Once that point was reached, the continuity of living cells became autonomous, and self-perpetuating cycles of life were underway [57].

Given the variability of the molecular constituents that likely would have characterized protocells from the beginning, it was inevitable that some variation existed in the molecular populations that got passed on to the descendant cells. Some components, structures, and functions based on the particular combination of metabolites inherited by some replicated cells would have been more successful than others in promoting the survival of the daughter cells from the beginning of fully autonomous cellular replication. Those cells that survived in greater numbers long enough to produce the next generation of daughter cells would have generated a larger proportion of daughter cells that contained the more favorable collection of metabolites, and the instruction for their synthesis. The earliest instances of natural selection would have been brought into play at that point.

#### 5.2.2. What Is Selected by Natural Selection?

In the classic concept of evolution through natural selection advanced by Charles Darwin [58], the unit of natural selection was the individual organism. Those organisms that chance variations endowed with a greater likelihood of survival to maturity were more likely to reproduce than other members of their species not similarly endowed. In the neo-Darwinian formulations of the early 20th century, emphasis shifted to the population gene pool as the level at which natural selection could best be described [59,60]. Those genetic variants that conferred an adaptive advantage were more likely to increase proportionately in the entire gene pool over time. As the molecular basis of genetic information was revealed in the second half of the 20th century, views about the unit of natural selection became blurred, with emphasis ranging from small segments of specific genes to whole taxonomic units at the species, or even higher, level [61]. Regardless of the focus of the unit of natural selection, what they all share is the fact that some biological features are more likely to survive long enough for the informational program that directs their construction and function to be passed along in greater proportion to succeeding generations. Perpetuation through reproductive success, for whatever reason, is the ultimate arbiter of which changes persist through time in the living world [14].

Sharma and Annila [12] have argued that “the rate of entropy production by various mechanisms is the fitness criterion of natural selection.” While I maintain that the fitness criterion of natural selection has to be reproductive success, I take Annila’s deeper meaning to be that natural selection leads to reproductive success by favoring those organisms that can maximize the entropy of the overall system of which they are a part. Annila goes beyond previous authors in relating fitness to the degree to which organisms *degrade energy most effectively*, in accordance with the PLA. An organism that can derive more energy from a unit mass of resources more quickly or efficiently has an advantage over its competitors, and therefore is favored by natural selection. From this perspective, the answer to the question of *why* evolution occurs in the living world is that natural selection favors adaptations that make ever more efficient uses of energy, thereby maximizing its degradation and increasing entropy overall.

This may explain in thermodynamic terms the engine that powers the evolution of life. However, an explanation of how and why these manifestations of the SLT and the PLA lead to proliferation over time of diverse forms of life, some of which become increasingly complex, requires some elaboration. A consideration of the major episodes in the history of life on our planet provides relevant illustrations.

#### 5.2.3. Major Transitions in the History of Life on Earth

The course of evolutionary change on Earth has been more like a series of cascades than a smooth flowing stream. The concept of punctuated equilibrium originated and popularized by Niles Eldredge and Stephen J. Gould [62] is the scientific version of this metaphor, and it dominates contemporary thought about the tempo of evolution. It also accords well with predictions of how evolution will proceed within the MEPP framework [23]. Among the notable transitions over the history of life on Earth that marked the onset of significantly increased degrees of energy dissipation are the following:

*Unicellular Chemotrophs*. The first reliably functioning, consistently replicating cells subsisted on chemical energy from their environment, mainly by extracting hydrogen (H) from an inorganic source for transfer to molecules with lower chemical potential energy, while capturing the energy difference in the form of high-energy compounds like adenosine triphosphate (ATP) and nicotinamide adenine dinucleotide (NADH). Not only did this retain the energy in a more useful form than just heat, it retained the energy until it was needed, and thus could be used more effectively by the cell. The second use of H was to reduce carbon dioxide (CO_2_) for producing the organic compounds (-CH*_n_*) on which living processes rely. The metabolic machinery for carrying out these reactions must have emerged at the earliest stages of life, possibly from elaborately specified mineral configurations that were taken up and improved upon by organic macromolecular catalysts [63,64]. Some of these early cells also developed the ability to extract hydrogen from organic compounds like methane (CH_4_). The end point of these energy-yielding pathways was primarily organic acids, like pyruvate and acetate, and alcohols, like ethanol. The earliest true cells had no internal membranes or nuclei, and hence were *prokaryotes* (“lacking nuclei”). Their architecture was simple and microscopic, but they markedly increased the transformation of energy. While physical planetary processes were using energy at an average rate of 75 ergs s^−1^g^−1^, the simple prokaryotic cell has been estimated to process around 200 ergs s^−1^g^−1^ [1].

*Unicellular Phototrophs*. Early in the history of life, certain unicellular autotrophs developed the ability to harvest the virtually inexhaustible energy of sunlight for producing their own food. Specialized protein–metal complexes like chlorophyll evolved as molecules with loosely held electrons that could be bumped to higher energy levels by absorbing photons of light. As they fell back to their ground state, the electrons gave up energy that could be captured to generate ATP and NADPH. The most elementary photosynthetic mechanisms may even have evolved from clays that provided photochemical machinery for making a few metastable molecules, such as polyphosphates, very simple carbohydrates, and ammonia [46]. However, once the sources of H became depleted, a more sophisticated photosynthetic mechanism then evolved that could couple electron excitation with the removal of H from H_2_O, a nearly ubiquitous resource, releasing free oxygen [48]. Absorption of photons and the splitting of water degraded the energy from the sun and increased entropy. Living cells became a new way to dissipate the ubiquitous solar energy impinging upon the Earth. Now, the energy of sunlight could be degraded by mechanisms within living cells that used it to build up organic molecules at the expense of a large increase in entropy and a substantially more effective use of solar energy.

*Oxidative metabolism*. Oxygen is very reactive, and the first thing it did upon being released from photosynthesizing cells was to oxidize whatever substrates were available. Once all the minerals that could be oxidized had reacted with oxygen, the oxygen left over began to accumulate in the atmosphere. It was a potent toxin to early forms of life, but natural selection enabled a few types of cells to survive through their ability to detoxify the oxygen by reducing it back to water [65]. A side benefit was the fact that water has a much lower chemical potential energy than does pyruvate, lactate, or ethanol, the end points of metabolism in the absence of oxygen. That meant that oxidation of H all the way down to water released much more total energy than was possible with organic compounds as the final H acceptors. This is the essence and revolutionary significance of oxidative metabolism [66]. Except for a few animal groups with very high metabolic rates, like mammals and birds, most organisms can survive for long periods of time without calling on the high energy yield of oxidative metabolism [67]. The metabolic process of glycolysis, for instance, converts one molecule of glucose to two molecules of pyruvate, releasing enough energy to provide a net gain of two energy-storing molecules of ATP. However, if oxygen is present, most organisms can further degrade pyruvate all the way down to six molecules of CO_2_ and up to 36 molecules of ATP, or 18 times the amount of energy that can be obtained by glycolysis alone [68]. The ability of oxidative metabolism to extract the maximum amount of energy possible clearly provides an adaptive advantage consequent to relentless pressure from the PLA.

*Unicellular eukaryotes*. For about half of the entire history of life on Earth, they persisted in the form of simple, prokaryotic cells. Evolution appears to have been “frozen” at the simple cellular level for close to two billion years. Many variations of the theme of these simple cells came and went, as natural selection optimized the mechanisms for energy degradation and self-replication. In time, however, larger and more complex cells arose through a series of revolutionary innovations [69]: Those included *enclosure of genetic material (DNA) within a membrane-bounded vesicle*, resulting in a primordial nucleus (eukaryote = “true nucleus”); *the formation of an internal cytoskeleton*, to provide structural stability; *folding of external membranes*, to increase surface area for nutrient absorption; *pinching off of enfolded membranes*, forming enclosed internal compartments, or organelles, with specialized metabolic functions; the *formation of digestive vesicles*, where larger molecules could be broken down; *endocytosis (engulfing) of prokaryotes with specialized metabolic capabilities* by another prokaryote lacking those metabolic processes; the *formation of cilia and flagella*, enabling the onset of cellular motility; and *evolution of mechanisms for replicating and segregating genetic information*, now localized in pairs of linear chromosomes, through their attachment to *a spindle apparatus* for segregating a member of each pair to enable movement to opposite poles prior to cell division.

The average eukaryote was three orders of magnitude larger and much more complex than a prokaryote. The innovations described above enabled the execution of a much greater number and variety of living processes, including the consumption and degradation of five times more energy per biomass by the simplest eukaryote (yeast) than a prokaryote like a bacterium [6].

*Multicellularity*. The transition from free living individual cells to cells clustered together for the life of the organism probably originated when dividing unicellular organisms simply failed to separate. At a subsequent stage, subgroups of cells which tended to specialize in certain functions provided the group with more effective and efficient means of carrying out processes vital for survival. Larger organisms with body parts specialized for optimal use of energy at different stages of their life cycle were far more effective at consuming and dissipating energy than were free living, mostly microscopic, individual cells. Metabolic rates, as measured by O_2_ consumption per mass of organism per unit of time, are roughly an order of magnitude greater in multicellular than in unicellular organisms [70].

#### 5.2.4. Evolution of Diversity in Living World

The evolution of eukaryotes from multiple combinations of specialized prokaryotes gave rise to many different types of eukaryotic cells. Some could produce their own food by photosynthesis (*autotroph =* “self-feeding”), and became something like modern green algae. Others good at oxidative metabolism but not at photosynthesis could efficiently extract energy from other organic compounds (*heterotroph =* “other feeding”) from their environment. Most biologists today recognize three kingdoms that evolved from unicellular eukaryotes. Plants arose from autotrophs. Fungi evolved from heterotrophs that absorb raw nutrients from their environment. Animals evolved from heterotrophs that ingest their food and digest it internally. Within each kingdom, great diversity eventually arose [57].

Animals are multicellular heterotrophs. They began appearing in the fossil record between 600 and 800 million years ago in three forms [71]. First, the sponges were multicellular, ciliated organisms arranged into stationary colonies of cells with internally directed cilia that drew seawater and its contents into the animal’s interior, where food particles could be digested. Secondly, cnidarians like jellyfish and sea anemones developed as radially symmetrical bodies with filamentous or arm-like extensions for capturing passing bits of living or dead nutrients and drawing them into the organism’s interior for digestion. Thirdly, worms emerged with the ability to ingest food from the substrate in which they lived. By 600 million years ago (Mya), worms had begun to evolve into a variety of body forms, which included appendages for grasping and locomotion, as well as antennae and other sensory organs. About 540 Mya, the “Cambrian Explosion” gave rise to a multiplicity of odd body forms and shapes, many of which did not survive while others became the templates for all the major animal phyla which persist to the present day [72].

The origins of biodiversity would appear to be straightforward: if life had a singular, simple origin, the factors that cause change and create diversity over time necessarily resulted in greater variety of forms with increasing complexity because those were the only directions possible [24,73]. Though the assertion that diversity has increased in the living world since life began would appear to be self-evident, the facts paint a more nuanced picture. Diversification has not always and inexorably increased. Since there is little fossil evidence pertaining to the first 2.5 billion years of life on Earth, little can be said with assurance about the diversity of the microbial life in ancient seas. Once fossils did begin to accumulate about a billion years ago, however, the course of biodiversity can be documented in a number of categories.

**Figure 3:**
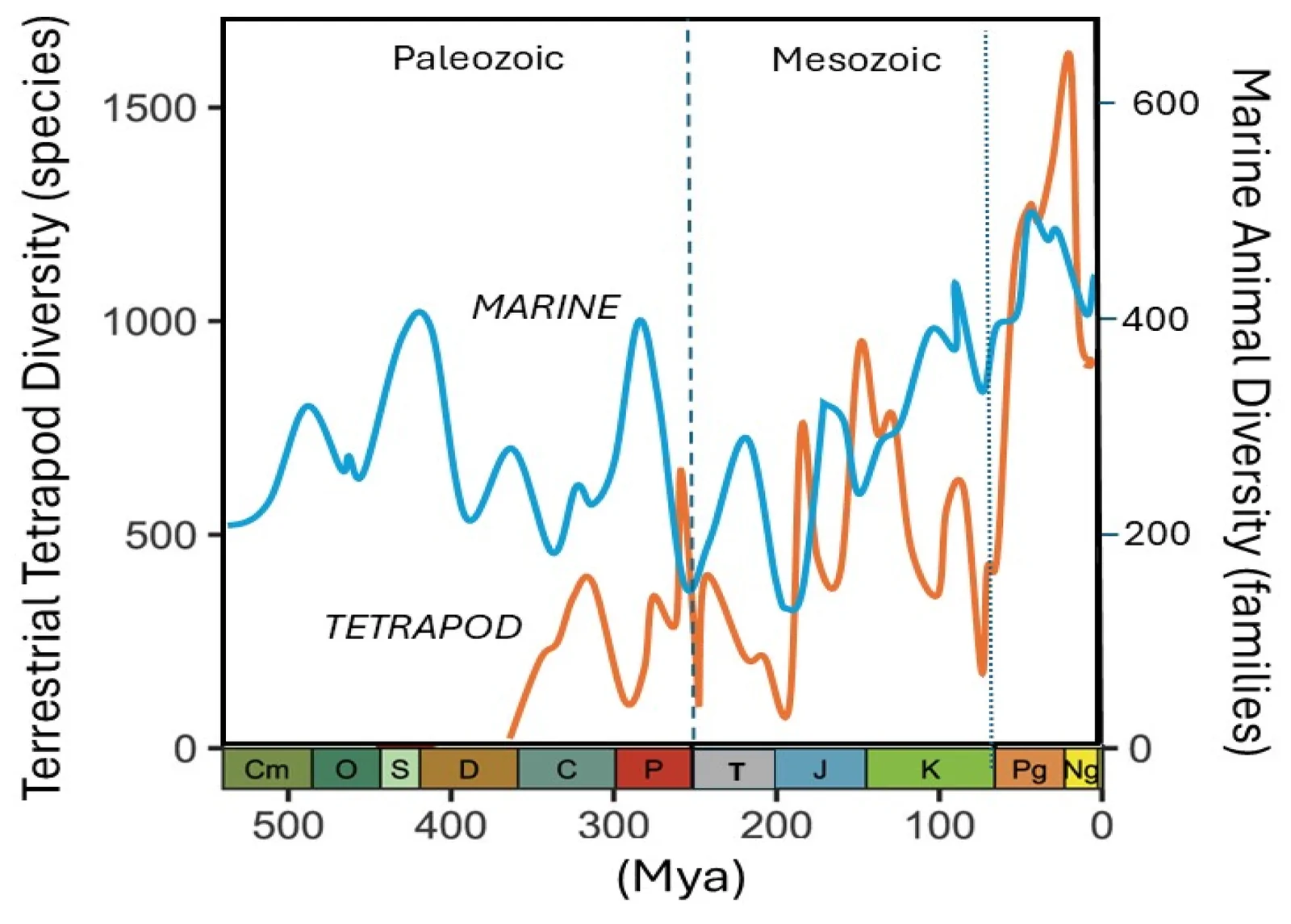
The biodiversity of marine animals (blue) and terrestrial tetrapods (orange) over time (Mya = million years ago), based on number of tetrapod species or marine animal families in the fossil record. The Cambrian (C), Ordovician (O), Silurian (S), Devonian (D), Carboniferous (C), and Permian (P) Periods comprised the Paleozoic Era. The Triassic, Jurassic, and Cretaceous Periods made up the Mesozoic Era. The Cenozoic Era (the last 65 million years) consists of the Paleogene (Pg) and Neogene (Ng) Periods. *Based on data from Alroy [74] for marine animals and Close et al. [76] for tetrapods*.

Following a steady but erratic increase for over a 250 million years, the number of different families of marine organisms peaked in the Permian, then declined to a low point at the Permian–Triassic (P-T) boundary, known to be the greatest extinction event in the history of life (Figure 3). Marine biodiversity started to rise again though erratically after that, but was slowed with a second extinction event, at the Cretaceous-Tertiary (K-T) boundary, before resuming another but decelerating increase [74].

Terrestrial plants evolved from marine aquatic photosynthetic ancestors, and obtained a foothold on the land during the Silurian Period [71]. By the start of the Devonian, the first ferns (Pteridophytes) were diversifying across the land in a steady increase until the late Permian, when the aforementioned crisis caused their number of species, along with those of their descendant conifers (Gymnosperms), to crash. After the P-T transition, conifers began to recover, but the ferns never again regained their peak diversity. Once the flowering plants (Angiosperms) evolved, they grew in diversity as the variety of conifers declined. The diversity of non-flowering plants actually peaked in the early Permian, never to be equaled again [75].

**Figure 4:**
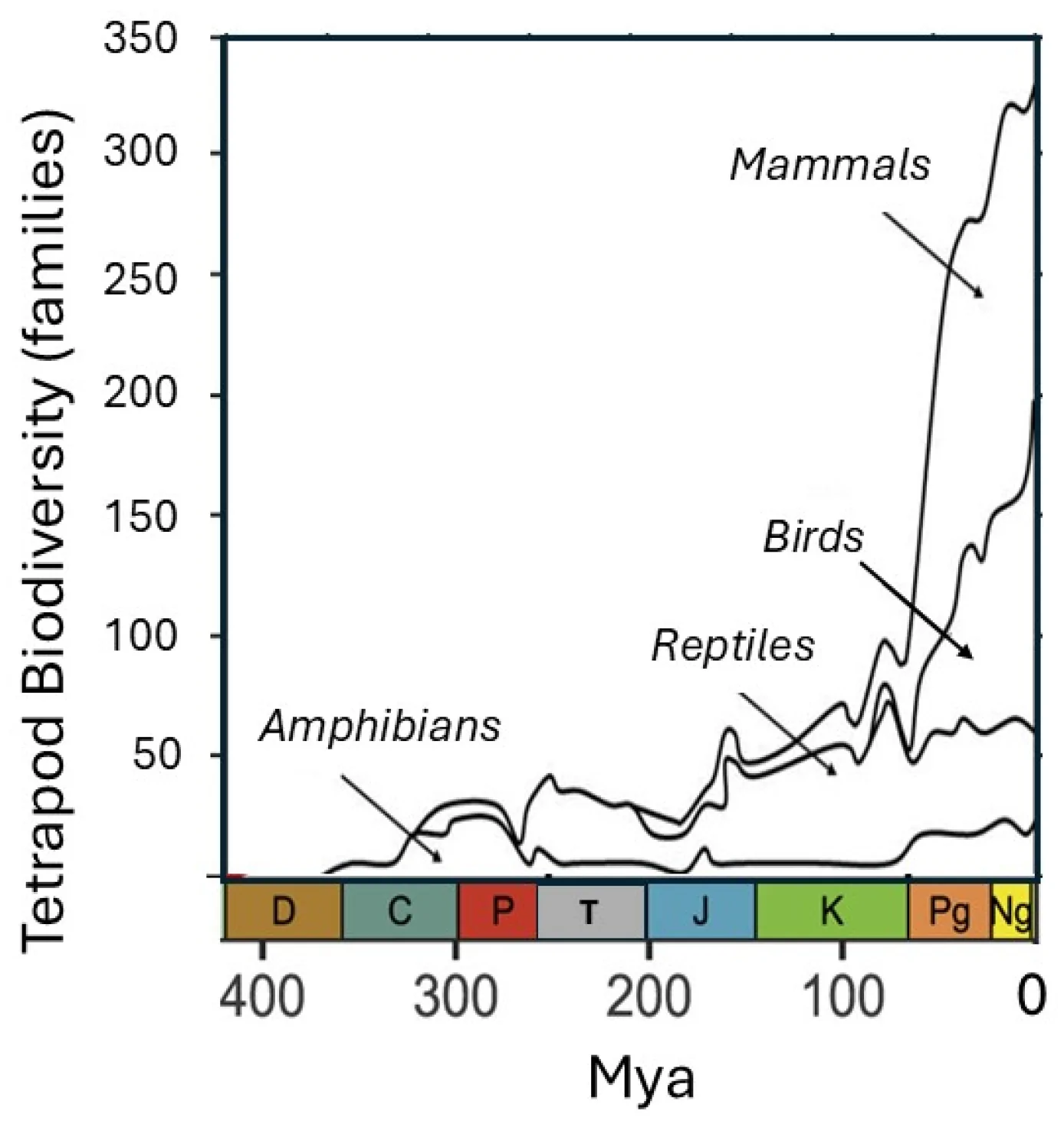
Biodiversity of amphibians, reptiles, birds, and mammals over time (Mya = million years ago). Geological ages as defined in Figure 3. *Based on data from Flegr [78]*.

The first animals on land were arthropods, dominated by insects [71]. The biodiversity of terrestrial tetrapods has risen progressively, though with periodic declines, throughout the Paleozoic and Mesozoic (Figure 3). Birds evolved from reptiles during the Jurassic but, like mammals, showed little increase in diversity until after the flying reptiles disappeared at the K-T boundary (Figure 4). Amphibians were the first vertebrates to diversify on land [77], beginning in the early Devonian. Their slow acceleration in diversity was superseded by reptiles, which first appeared during the Carboniferous, prior to the crash at the P-T boundary. Reptiles recovered during the Triassic, but amphibians never did. Reptilian diversity remained essentially level, as that of mammals vastly accelerated following the K-T extinction that ended the dominance of the dinosaurs [76].

As this brief review reveals, biodiversity has shown a demonstrable net increase over time, but has been anything but steady and inexorable. Quite often, diversity has reached a steady state that is maintained for many millions of years, as for marine animals from the early Devonian to the late Permian (Figure 3), and for reptiles from the late Jurassic through the Cretaceous (Figure 4). Dramatic increases in diversity most often have occurred after ecological crises, perhaps precipitated by geophysical or meteorological catastrophes, leading to mass extinctions. Very often an increase in the diversity of one group supersedes the decline in another, as when conifers took over from ferns, and mammals eclipsed reptiles (Figure 4). Occasionally, however, diversity among different groups changes in tandem, as shown by insects and birds during the Cenozoic after flying reptiles had disappeared. In summary, while overall biodiversity does tend to increase over time, diversification within groups is episodic and dependent on ecological constraints, both among different groups of organisms, and between organisms and their environments.

#### 5.2.5. Evolution of Complexity in Living World

Complexity, in Neubauer’s [5] straightforward use of the term, is taken to mean the number of parts and the number of functional relationships between those parts. Complexity can be viewed in structural, functional, or algorithmic terms. Since the origin of the universe, complexity has tended to increase primarily in structural (physical), then functional (biological), and then algorithmic (social) forms, in that sequence [8]. But every system, from atomic structure to economics, has both structural and functional aspects, and at least a theoretical algorithmic definition.

If life began, as commonly assumed, in the form of simple solitary cells which diversified over time, it stands to reason that, as more forms evolved, some of them would logically have to have become more complex if life truly was in the simplest form possible in the beginning [23,79]. A comparison of Earth’s biosphere today with what it must have been like 3.5 billion years ago leaves no doubt that life has evolved into increasingly complex forms over time. But like diversity, empirical observations provide a nuanced picture of evolving complexity, and some unexpected observations.

**Figure 5:**
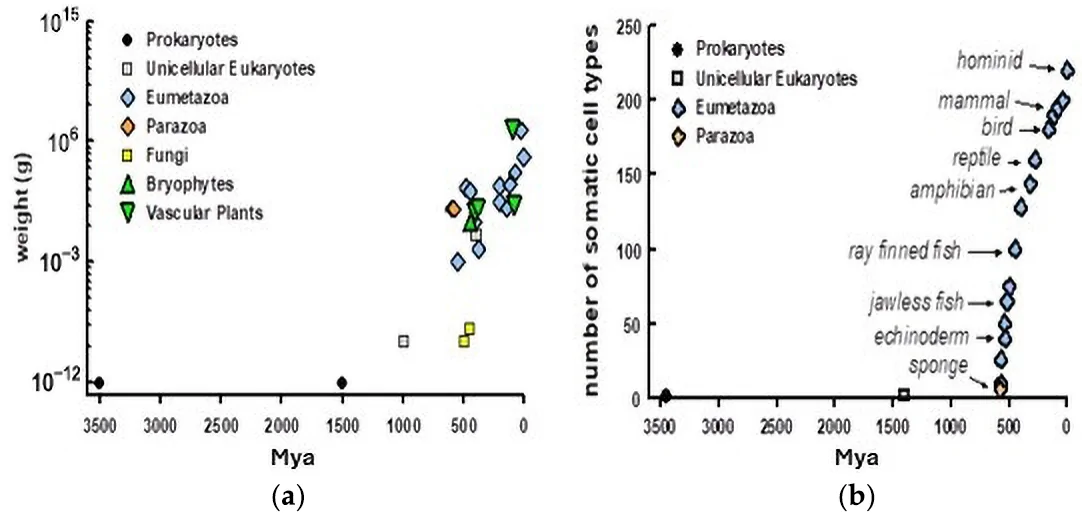
The rise in structural complexity over evolutionary time: (**a**) organismic size; (**b**) the number of different somatic cell types (exclusive of neurons) per organism. The time point in million years ago (Mya) plotted for each organism coincides with the appearance of its ancestral form. Bryophytes are the mosses. Parazoa are the sponges. Metazoa are all multicellular animals other than sponges. *Data on size from Tekwa et al. [80]*; *data on somatic cell number from Valentine [72]*.

Using growth in size as a simplistic proxy for structural complexity [8], the increase in organismic size over evolutionary time is readily evident. From a simple prokaryotic cell over 3 billion years ago to the largest organism extant today, size has increased by about 19 orders of magnitude (Figure 5a). The vast majority of that increase, however, has occurred within the last one-third of the history of life. The effect is shown even more dramatically by plotting the number of different cell types―a better measure of complexity as defined above―which have evolved in the animal kingdom over the same period (Figure 5b).

**Figure 6:**
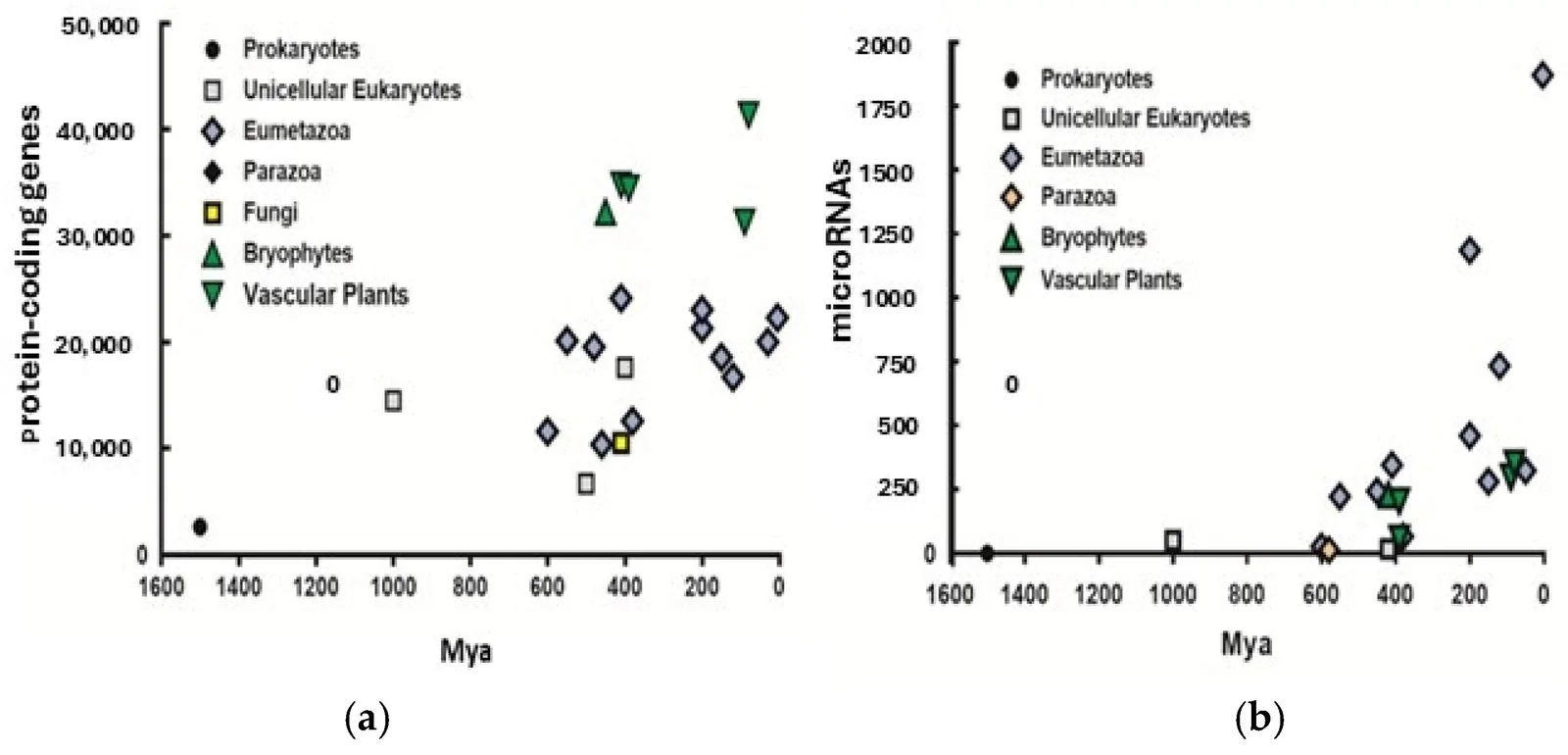
The rise in functional complexity over evolutionary time: (**a**) the number of protein-coding genes per organism; (**b**) the number of regulatory genes as measured by microRNA transcripts per organism. The time point in million years ago (Mya) plotted for each organism marks the appearance of its ancestral type. Various organisms were selected on the basis of data availability. *Data from https://www.ensembl.org/ for protein coding genes, and https://mirbase.org for microRNAs*.

Multicellularity beyond colonial aggregation gave rise to cellular differentiation. When this became a feature of the organism’s life history, a developmental time course became a definitive characteristic. Thus, programming for developmental transitions had to become part of the genetic endowment of each species. This is reflected by the fact that information for the construction and function of each stage of the life cycle requires larger genomes in multicellular than in unicellular organisms. Since proteins carry out the functions of the cell, the number of different proteins coded is one way to measure functional complexity. That plot (Figure 6a) shows a progressive increase, especially among the eumetazoans, that corresponds to the onset of multicellularity about 600 million years ago.

Note that the greatest number of protein-coding genes is seen in plants, with flowering plants being the most complex organisms ever evolved by this criterion. Some of this can be explained by the tendency of plants to retain multiple copies of genetic information because of the way they reproduce, resulting in multiple possibilities for the mutational origin of new protein-coding genes. If they survive elimination by natural selection, they presumably have a function. This suggests that in sessile organisms, more diverse protein chemistry and metabolism may compensate for the organism’s inability to move about in its environment.

Another measure of functional complexity is the number of regulatory genes that affect differential gene expression (Figure 6b) [5].

In summary, complexity, like diversity, has shown a net increase over evolutionary time, but in nothing like a linear fashion. For two billion years, the level of complexity of living organisms remained basically static. Then, between a billion and 500 Mya, complexity began to increase dramatically. Because this coincides so closely with the rise of multicellularity, the increase in both complexity and diversity most likely is related to that epic transition in the history of life.

To what can the overall increase in organismic complexity be ascribed? I agree with those authors who argue that biodiversity tends to lead toward a net increase in complexity [8,35,79,81], and that complexity has evolved over time to act as an increasingly effective adaptive system for resolving thermodynamic gradients.

This section has emphasized the “progressive” nature of organic evolution in which both diversity and complexity show net increases over time. It is important to emphasize, however, that increases in neither diversity nor complexity are inevitable for any single group of organisms. George Gaylord Simpson pioneered the realization that different groups of organisms evolve at different rates and that long periods of stasis are common [82]. Evolution is cyclic, in that existing forms diversify under the pressure of directional selection, mature to static forms under stabilizing selection, and eventually decline and go extinct for a variety of reasons [14]. The focus of this essay, however, is to explain why diversity and complexity show an overall net increase over evolutionary time.

The thermodynamic argument goes thusly: The expanding universe leaves in its wake local inhomogeneities of matter and energy [1]. These result in gradients that cause the flow of matter and energy from sources to sinks. The flow of energy through matter tends to organize that matter into adaptive systems for dissipating the energy gradients [35,56]. Stochastic processes, such as mutations of the genetic repository of organismic information and other emergent events like morphogenetic innovation [83], generate diversity, and that diversity inevitably leads to some adaptive systems that are more complex than others [84]. Those adaptive systems which can resolve energy gradients more effectively tend to be more complex [6,7,57,85,86], and therefore enjoy a selective advantage, although an intermediate rather than extreme level of complexity is often more effective [8,87,88]. A more precise statement may be that adaptive systems evolve greater complexity to the extent that the increased complexity dissipates more energy per unit of time and organismic mass. Either way, the thermodynamic point of view can be summarized by saying that living organisms are complex adaptive systems that have evolved in a way that progressively tends to dissipate more energy [1,5,6,7,12,21,84,89].

The degree or energy dissipation enabled by entities at different orders of magnitude in the physical and living world is shown in Table 1. Energy dissipation by entities on a cosmic scale (galaxies, stars, planets) is governed by means of gravity, radioactive decay, and electromagnetic radiation or absorption, while living organisms on Earth process energy that originates primarily as sunlight.

At this point, the matter of perspective is worth considering. Is the egg a chicken’s way of making another chicken, or is the chicken an egg’s way of making another egg? From the perspective of living organisms, natural selection is the means by which adaptations are improved and new species are formed. From the perspective of the energy content of the universe, organic evolution is the means for bringing about changes that use energy and increase entropy more effectively. Both statements are factually defensible.

Great progress has thus been made in reconciling the evolution of life, which has tended to lead to increasingly complex organisms over time, with the SLT’s imperative that free energy be degraded and entropy be increased overall in the organism and its environment. Notwithstanding the impressive way in which scientists have succeeded in applying the SLT and PLA to the evolutionary process, the actual time course over which diversity and complexity have increased in the living world does not lend itself to easy explanation. The universe has been expanding, and entropy has been increasing steadily over the entire history of life on Earth. There would appear to be no obvious way to explain why expansion and entropy could have so little effect for two billion years of relatively static and simple prokaryotic life, then generate one revolutionary innovation―the eukaryotic cell―about a billion and a half years ago, followed by an explosive increase in diversity and complexity over only the most recent 600 million years. Part of the explanation probably lies in the fact that organic evolution is subject to a variety of proximate influences, including many that do not directly involve energy. Those include reproductive strategies and sexual selection, ecological contingencies, geophysical catastrophes, genetic drift, and historical constraints. The concepts of punctuated equilibrium [62] and frozen evolution [78] are probably relevant, and some very significant threshold effects are likely at play. Evolution of the eukaryotic cell was clearly one such threshold. The emergence of multicellularity was another. In both cases, structural complexity increased dramatically (Figure 5), and with it a significant increase in functional and algorithmic complexity (Figure 6) was seen. This point will be considered further in Section 6.

### 5.3. Evolution of Brain and Behavior

Another threshold was reached when animals evolved with the ability to move about in their environments, seeking food and mates, including the pursuit of and flight from one another. This required real-time information about the environment, and control of coordinated body movements for locomotion. Pressured by the need for analysis, management, and storage of large amounts of dynamic information, nervous systems evolved, enabling the behavior of organisms, capable of altering their environments and interacting with one another. At this point, neural information became an added form of algorithmic complexity, supplementing genetic information with far reaching consequences for the evolution of life.

Behavior is inherently adaptive, since natural selection favors behaviors that promote survival. As indicated above, selective survival and reproduction are distinctive features of biological evolution, and therefore critical to the greater degree of energy dissipation living organisms bring about.

#### 5.3.1. Evolution of Nervous Systems

In its architecture and function, the nervous system is the most complex organ of an animal, and―not incidentally―the most energetically demanding per unit of mass. The end result of the evolution of nervous systems has been the capacity for animals to become active participants in the web of life, beyond the passive role played by the other kingdoms that occupy the biosphere.

Inasmuch as animals in general have evolved into larger and more complex forms over time, so too, on average, have the nervous systems that serve to integrate sensorimotor information, regulate their physiology, and control their behavior. Notable advances in neural complexity are seen in the evolution of flatworms, then annelids and arthropods in one direction and mollusks in the other [90]. Within each of these groups, a few have developed much more complex and integrated nervous systems than others, while the majority have remained similar to that of their simpler ancestors. Among the mollusks, the most spectacular increment in size and complexity is seen in the cephalopods (squid, octopods, and cuttlefish), whose brains and behavior rival those of some vertebrates [91].

The vertebrates, in turn, show a great variety of neural complexity in all groups. The ancestral forms arose in the late Cambrian, probably from free-swimming larval forms of invertebrates [71]. They proliferated, first as jawless then as jawed fish in Ordovician oceans. Their active locomotion and need to analyze rapidly changing sensory information in real time favored the evolution of complex nervous systems. When amphibians evolved, probably from lobe-finned fish with lungs in the mid-Devonian, about 370 Mya [77], they retained the basic organization of the nervous system and brain anatomy of their ocean-dwelling ancestors, though in some―most notably the lungfish and salamanders―that neural complexity has clearly regressed [92,93]. Reptiles evolved from amphibians. Their total freedom from an aquatic environment, combined with a generally more active lifestyle, imposed the need for more complex neural coordination and a larger brain.

Mammals evolved from reptiles during the early Mesozoic, over 200 Mya. Birds evolved independently from a separate reptilian line some 30 million years later [94]. Both groups are characterized by having significantly larger brain-to-body mass ratios than do all other vertebrates. When mammals first arose, the daytime niches were filled with ruling reptiles. The nights were safer for the smaller vulnerable mammals, but were cooler and devoid of light. Mammals thus needed to stabilize their body temperatures at a higher level (homeothermy) in order to remain mobile, and had to depend on non-visual senses, especially olfaction and hearing, for information about their environments. Olfaction and hearing provide qualitative information requiring memory and its related faculty, a good sense of temporal ordering, to be effective. While birds, occupying mainly daytime niches, continued to rely heavily on vision, their dynamic activity in three-dimensional space required a higher degree of coordination between high-definition visual input and real-time sensorimotor adjustments, as well as the high energy consumption that flight demanded. Together, these nervous system innovations in birds and mammals supported a life style involving a significant increase in energy utilization over that required by reptiles.

#### 5.3.2. Brain Evolution in Hominins

Relative brain size and complexity have increased most spectacularly in humans, but this is a recent event compared to similar changes that appeared independently about 20 million years earlier in psittaciformes (parrots), corvids (ravens and crows) and cetaceans (dolphins and whales), not to mention over 400 million years ago in cephalopods.

Elephants and whales have the largest brains of any contemporary animal, but humans have the largest brain-to-body mass ratio by far. Primates as a group have larger brains than would be predicted by their body sizes; however, the hominin line leading to modern humans began to exceed in relative brain size that of the other great apes when australopithecines evolved about four million years ago [95]. This was followed by two dramatic increments in relative brain size. The first occurred a little over two million years ago when *Homo habilis* split off from its ancestral genus, *Australopithecus* [96]; the second came when *Homo sapiens* emerged about 300,000 years ago [97]. For the two million years between *Homo habilis* and the extinction of *Homo erectus*, relative brain size increased very little, and has remained in a plateau since the final burst in relative size (largely attributable to a decrease in body size) over the last 100,000 years. The human brain does not differ in gross anatomy from that of its closest living relative, the chimpanzee, but it does have a few novel organizational features [95,98]. These include expansion of the dorsal temporal lobe, which processes auditory information related to speech, and expansion of regions of the parietal lobe, which include somatosensory cortex [99]. In summary, the human brain has become relatively larger, and through organizational innovations, more complex, over evolutionary time.

#### 5.3.3. Evolution of Cognitive Capacity

When nervous systems emerged in the course of evolution, they enabled organisms to become engaged with their environments in more diverse and complex ways. To increasing degrees, animals were freed from the immediate constraints of their surroundings. Sensory input gave them an internal connection with their external world. Motor coordination gave them the capacity to resist the forces impinging upon them, generate new ones to the point of changing their environment, or at least their situation within it. Thus, animals were able to become increasingly and proactively independent actors in the ecosystems of which they were a part.

To the extent that disengagement from the environment was a positive adaptation, it was restricted by natural selection in ways that favored survival. Thus, not all sensory input that the environment could provide was relevant to a given animal in a particular situation. Color vision was of no use to a nocturnal animal, so neural architecture otherwise devoted to that would be better spent on a high degree of olfactory sensitivity. Not all motor capabilities were equally useful; speed was more important than strength for small prey, while stealth and strength better served the predator. As natural selection honed the sensory abilities and motor capacities of each species according to the niche each occupied, patterns of information processing and control were programmed into the nervous system of each that molded the animal’s ability to navigate through its daily life. Those patterns of information processing by definition were species-specific: to optimize the animal’s continued survival and contribution to energy dissipation.

There has been an unquestionably explosive growth in the human brain since the hominid divergence from other primates [99,100]. This has been attributed to a host of factors encompassing the “human adaptive complex” of increased socialization, mastery of fire and tool use, reproductive strategies, and life history adjustments [14,101]. As brains got larger, changes in diet, dentition, and digestive anatomy evolved to extract a greater yield of energy from food [96]. Specifically, the incorporation of more animal fat and protein into the human diet, along with morphological changes in dentition and the gastrointestinal tract promoted support of “energetically expensive” brain tissue [102]. Much earlier, a thermodynamic interpretation of the evolution of the human brain was offered by Kirkaldy [103]. Since most of the increase in brain size has been attributed to expansion of the forebrain, where insight and anticipatory analysis are centered, there has been a tendency to view anticipatory, analytical, problem-solving behavior as an endpoint favored by natural selection. While certainly one measure of cognitive advancement, this definition is clearly self-referential. The cognitive abilities of other animals with large and complex brains are largely unknown to us. The remarkable sensory–motor manipulative ability of cephalopods, the long-range communication skills and social habits of the cetaceans, and exquisite auditory sense and social memory of elephants extend to realms which we barely understand. Those capacities evolved over time through changes in the brains and nervous systems that have been unique to each of those groups. At the same time, most members of the animal kingdom have persisted at a plateau of neural complexity totally adequate for their needs, once the defining behaviors and nervous systems subserving them first emerged.

Behavior is an energy-consuming process. Some energy is expended in order to acquire more energy than can be extracted from the environment by remaining stationary. The short-term use of this energy is to promote the survival of the species within the strictures of its particular niche and lifestyle. Birds and mammals need a lot of energy just to maintain high body temperatures, enabling them to remain active at lower ambient temperatures for seeking mates and food, or keeping alert to danger. Predators need energy for chasing and capturing prey, as the latter need it for escaping. Animals build nests, dam rivers, dig burrows, and transport themselves by flying, swimming, walking, running, and hopping. Humans build houses and machines and toys, for shelter, work, and the social ends of play. All of these energy-consuming activities are geared toward the proximate aim of survival. And this brings us again to the fact that the arc of evolutionary history has followed the mandate of the PLA, degrading energy ever more efficiently and effectively in the direction required by the SLT.

## 6. Discussion

Any overarching hypothesis like the one proposed here that seeks to explain so much runs the risk of implying that it has stand-alone explanatory power. No such implication is my intention. The following are ways in which the energy dissipation model of the evolutionary imperative alone does not suffice to explain all aspects of change in the natural world.

Energy degradation itself is seldom the proximate cause of evolutionary change. Mountains form due to the collision of continental plates, and are eroded by rain and wind. Seasons change due to a planet’s orbital position and tilt relative to its central star. Life originated when organic reactions governed by chemical principles led to molecules with certain desirable properties. Photosynthesis likely arose to detoxify oxygen, then oxidative phosphorylation evolved to capture the energy from the breakdown of nutrients into manageable metabolic units. The plumage of a peacock, the mating call of a frog, and the territorial display of lizards are behavioral adaptations for promoting reproductive success. In all of these cases, the dissipation of energy is an ultimate outcome, but the proximate cause can be argued to be plate tectonics, orbital mechanics, cellular toxicity, intermediary metabolism, or natural selection, as the case may be.

While the SLT and PLA affect the direction and pathway of changes over time, the specific changes that actually occur are subject to a number of constraints. Some of these are developmental, since a pre-existing developmental program of an organism represents a starting point for all the evolutionary changes that follow. For example, morphological changes affected by developmental constraints suggest that fetal mass is the principal determining factor in setting limits to the length of gestation [104]; morphogenetic processes taking place during embryogenesis play a decisive role in evolutionary changes in the nasal skull of cetaceans [105]; and vestigial organs reflect developmental loss of previously functional body parts [93,106,107].

Cellular constraints were clearly critical for the independent onset of multicellularity in each kingdom of life [108], as that innovation depended on the prior development of mechanisms for cell adhesion.

Another evolutionary constraint is behavioral. For example, the evolution of reproductive behavioral complexity in fiddler crabs may have arisen multiple times during their evolution by coopting a series of other adaptations for high intertidal living and antipredator escape [109]. Another example is evidence that species which share similarities in locomotor behavior, mode of prey capture or cognitive ability share similar brain organization [110].

Ecological constraints have been an obvious influence on adaptive evolution. Examples include the mid-Cretaceous radiation of ants in forest ground litter and soil coincident with the rise in flowering plants [111], the higher survival rate of megafauna during the late Pleistocene by species occupying arboreal or nocturnal habitats despite having low reproductive rates [112], and the positive correlation between the evolution of larger brains with habitat complexity [113]. A mixture of ecological and behavioral constraints appears to have influenced the divergence of humans from the other hominids [114].

A fair question to ask about the evolutionary imperative is why, if evolution is imperative, is it not constantly occurring? The sun has been burning for about 4.5 billion years without significant change other that a depletion of hydrogen fuel and a gradual increase in brightness. For the first two billion years of the history of life, organisms remained unicellular and relatively simple [69]. Tetrapods invaded the land over 400 Mya and evolved the muscles and bones for their limbs that have remained remarkably constant to the present time [94]. Humans have the same three bones in their hindlimbs―a femur, tibia, and fibula―as dinosaurs.

A related question is why, if the evolution of complexity better serves to consume and dissipate energy, does it not occur inevitably instead of arresting at a static level or even regressing on occasion (see Section 5.2.5)? In the first place, several observers have noted that the PLA calls for an optimal (usually intermediate), not maximal, level of complexity for maximally efficient energy dissipation [8,56,87,88]. In the second place, evolution of new structural or functional complexity would usually entail greater energetic costs to enhance an optimal level of fitness that already exists for an organism in a static environment. Evolution appears to promote adaptations that are “just enough” to promote the survival of an organism in its given niche. A change in that niche (usually the environment) is needed to enlist the benefits of increased complexity.

In conclusion, I have sought to provide a concise summary of the energy dissipation model of the evolutionary imperative, excerpted from a more detailed treatise published previously [14]. Nothing other than the title for the model is claimed to be novel. My aim was to make available to a broader audience the highly technical work of Annila and his colleagues [4], and the related arguments by Neubauer [5] and Chaisson [1,6], along with many specific examples which support the model from the physical and living worlds.

A final caveat to the argument advanced here is obvious but needs to be acknowledged explicitly. It seems very likely that nature operates through some processes and principles of which we are still ignorant. The nature of dark matter, dark energy, and antigravity are obvious examples of our incomplete understanding of all the factors that govern the properties of the natural world. While I believe that the role of the SLT and PLA in channeling the arc of evolution can be advanced with confidence, to argue that it explains everything about the evolutionary imperative could well turn out to be naïve in the fullness of time.

## Figures and Tables

**Table 1:** Evolutionary increase in energy flux, measured as energy rate densities.

Entity	Ergs s^−1^ mg^−1^
*Protostar*	*0.001*
*Sun (current)*	*0.002*
*Earth*	*0.07*
Protists	0.9
Fish/Amphibians	4
Plants (Gymnosperms)	5
Plants (Angiosperms)	8
Reptiles	3
Mammals	40
Birds	70
Humans (hunter/gatherer)	40
Humans (agricultural worker)	100
Humans (industrial laborer)	500

Energy rate values are rough estimates for an average entity in each category, based on data from Chaisson [6]. Entities are listed in their approximate order of evolutionary appearance. Energy flux for entities in italics is dominated by the force of gravity, while in living organisms it traces ultimately to sunlight.

## Data Availability

Data on which the argument in this essay are based were found in the original publications cited.
